# Decreased expression of B7-H3 reduces the glycolytic capacity and sensitizes breast cancer cells to AKT/mTOR inhibitors

**DOI:** 10.18632/oncotarget.6902

**Published:** 2016-01-12

**Authors:** Caroline E. Nunes-Xavier, Karine Flem Karlsen, Christina Tekle, Cathrine Pedersen, Tove Øyjord, Vesa Hongisto, Jahn M. Nesland, Ming Tan, Kristine Kleivi Sahlberg, Øystein Fodstad

**Affiliations:** ^1^ Department of Tumor Biology, Institute for Cancer Research, Oslo University Hospital Radiumhospitalet, Oslo, Norway; ^2^ Department of Genetics, Institute for Cancer Research, Oslo University Hospital Radiumhospitalet, Oslo, Norway; ^3^ Misvik Biology Oy, Turku, Finland; ^4^ Department of Pathology, Oslo University Hospital Radiumhospitalet, Oslo, Norway; ^5^ Department of Oncologic Sciences, Mitchell Cancer Institute, University of South Alabama, Mobile, AL, USA; ^6^ Department of Research, Vestre Viken, Drammen, Norway; ^7^ K.G. Jebsen Centre for Breast Cancer Research, Institute for Clinical Medicine, Faculty of Medicine, University of Oslo, Oslo, Norway; ^8^ Institute for Clinical Medicine, Faculty of Medicine, University of Oslo, Oslo, Norway

**Keywords:** CD276/B7-H3, breast cancer cells, API-2, everolimus, glycolysis

## Abstract

B7 family proteins are important immune response regulators, and can mediate oncogenic signaling and cancer development. We have used human triple-negative breast cancer cell lines with different expression levels of B7-H3 to evaluate its effects on the sensitivity to 22 different anticancer compounds in a drug screen. API-2 (triciribidine) and everolimus (RAD-001), two inhibitors that target the PI3K/AKT/mTOR pathway, showed enhanced inhibition of cell viability and proliferation in B7-H3 knockdown tumor cells compared to their B7-H3 expressing counterparts. Similar inhibition was seen in control cells treated with an anti-B7-H3 monoclonal antibody. In B7-H3 overexpressing cells, the effects of the two drugs were reduced, supported also by *in vivo* experiments in which B7-H3 overexpressing xenografts were less sensitive to everolimus than control tumors. In API-2 and everolimus-treated B7-H3 overexpressing cells, phospho-mTOR levels were decreased. However, phosphorylation of p70S6K was differentially regulated in B7-H3 cells treated with API-2 or everolimus, suggesting a different B7-H3-mediated mechanism downstream of mTOR. Both API-2 and everolimus decreased the glycolysis of the cells, whereas knockdown of B7-H3 decreased and B7-H3 overexpression increased the glycolytic capacity. In conclusion, we have unveiled a previously unknown relationship between B7-H3 expression and glycolytic capacity in tumor cells, and found that B7-H3 confers resistance to API-2 and everolimus. The results provide novel insights into the function of B7-H3 in cancer, and suggest that targeting of B7-H3 may be a novel alternative to improve current anticancer therapies.

## INTRODUCTION

The B7 family of immune receptors is considered essential in the regulation of the adaptive immune system, but its members are also expressed in non-hemopoietic tissues and are emerging as important players in cancer [1]. These proteins can be divided into three groups, according to the signals they transduce during T cell activation: I) co-stimulatory (e.g. B7-1 and B7-2); II) inhibitory (e.g. B7-H1); and III) co-stimulatory/inhibitory (e.g. B7-H3 and B7-H4) [2, 3]. The B7 proteins are mostly expressed in the plasma membrane and interact with receptor partners in T and NK cells, which mediate their immune modulatory functions, but their partners in non-hemopoietic cells are mostly unknown [4–6].

B7-H3 is overexpressed in many types of carcinomas (including breast, colorectal, pancreas, prostate and lung cancer), and in sarcomas and melanomas [7–12], but is not expressed, or expressed at low levels, in most normal cells or tissues [13, 14]. Interestingly, a new breast cancer detection approach was recently reported by using B7-H3-targeted ultrasound molecular imaging [15]. It has been suggested that B7-H3 plays a role in immune evasion of breast cancer [16]. However, the precise molecular basis for the functional role of B7-H3 in cancer remains unclear. In this context, we have identified an oncogenic non-immunological role of B7-H3 in melanoma and breast cancer, which promotes metastasis and resistance to chemotherapy. These effects could be exerted through regulation of proteins in the JAK2/STAT3 signaling pathways, as well as by modulating the expression of cytokines and other metastasis-associated genes [12, 17].

Around 15% of all breast cancers do not express estrogen receptor, progesterone receptor, and epidermal growth factor receptor 2 (HER2/Neu), and are classified as triple-negative breast cancer (TNBC). This type of breast cancer is amongst the most aggressive types, with poor overall survival, mainly due to the lack of specific therapies. Thus, more efficient therapeutic options for TNBC patients are needed. B7-H3 expression is elevated in TNBC, and high expression has been associated with tumor progression and metastasis, as well as with shorter recurrence-free survival [18–22]. B7-H3 has emerged as a new potential target in TNBC and other cancer types, and its targeting by monoclonal and bispecific antibodies (MGA271; MacroGenics, MGD009; MacroGenics, H89; Memorial Sloan Kettering Cancer Center) are currently being studied in several phase I clinical trials in patients with B7-H3-positive cancers, including TNBC (ClinicalTrials.gov: NCT01391143, NCT0191893, NCT02 381314, NCT02475213, NCT01099644, NCT01502917, NCT00089245) [23, 24].

In this study, we have assessed the role of B7-H3 in the viability of human TNBC cell lines, and performed a drug screening to evaluate the effect of a panel of different anticancer compounds. We found that the effect of API-2 (triciribidine) and everolimus (RAD-001), two small molecule inhibitors targeting the PI3K/AKT/mTOR pathway, was partially dependent on B7-H3 expression. API-2 and everolimus, as well as decreased expression of B7-H3, reduced the rate of glycolysis. Our data support the notion that targeting B7-H3 may a valuable adjunctive approach to improve breast cancer therapy.

## RESULTS

### Decreased expression and inhibiton of B7-H3 increases the sensitivity of breast cancer cells to AKT/mTOR inhibitors API-2 and everolimus

**Figure 1:**
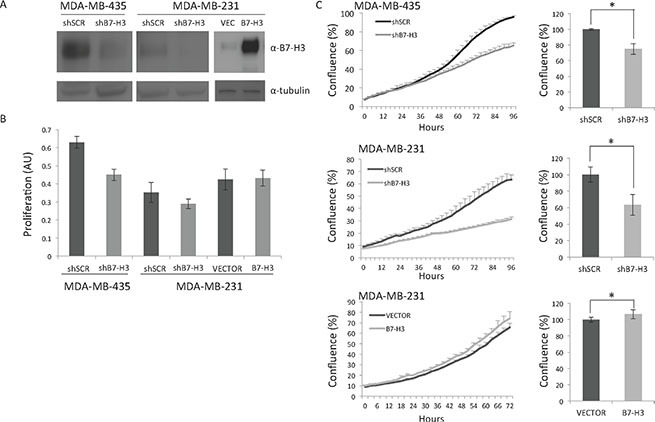
Immunoblot and proliferation analysis of the cell lines (**A**) Immunoblot of B7-H3 and tubulin expression from total lysates from MDA-MB-435 and MDA-MB-231 cell variants. shSCR, control short hairpin scrambled cells; shB7-H3, short hairpin B7-H3 cells; VEC, control vector cells; B7-H3, overexpressing B7-H3 cells.; α, anti. (**B**) Proliferation of the MDA-MB-435 and MDA-MB-231 cells. Proliferation was measured by the MTS assay after 3 days in culture ± S.D., Cell variants are as in A. (**C**) Left panels, cell confluence was measured growing the cells in IncuCyte ZOOM Kinetic Imaging System (Essen BioScience). Cells were scanned every three-hour during the times indicated. The data is presented as percent cell confluence ± S.D. Statistically significant results are marked with *Right panels, relative final confluence from two independent experiments is shown (top panel, *p = 9.87199E–15; middle panel, *p = 1.06099E–05;bottom panel, *p = 0.000702). Cells variants are as in A.

We have previously established MDA-MB-435 and MDA-MB-231 TNBC cell lines with stable knockdown of B7-H3 expression (shB7-H3) or scrambled (TR33 here referred to as shSCR) via short hairpin RNA (Figure 1A, left and middle panels), as described previously [17]. We have now also generated MDA-MB-231 cell lines stably overexpressing B7-H3 (Figure 1A, right panel). Growth, measured as cell confluence of parental, and control vector or shSCR cells was similar (Supplementary Figure S1). B7-H3 knockdown decreased cell proliferation as measured by MTS after 3 days of culture (Figure 1B, and as reported previously [17, 25]). Moreover, growth of shB7-H3 MDA-MB-453 and MDA-MB-231 cells, measured as cell confluence during 3–4 days was significantly decreased, whereas a slight significant increase was observed in B7-H3 cells (Figure 1C).

**Figure 2:**
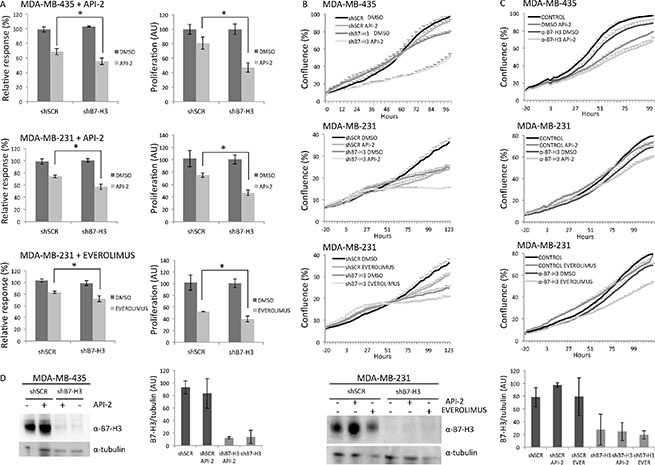
Effects on *in vitro* proliferation of MDA-MB-435 and MDA-MB-231 B7-H3 knockdown cells treated or not with API-2 and everolimus (**A**) Left panel, growth inhibition of the cells was measured by cell viability assay (CTG) after 5 days of treatment of cell variants: MDA-MB-435 shSCR and shB7-H3 cells with 1 mM API-2 (upper panel, *p = 0.00166) and MDA-MB-231 shSCR and shB7-H3 cells with 1 mM API-2 (middle panel, *p = 0.002208) and 10 μM everolimus (bottom panel, *p = 0.001515). ± S.D., statistically significant results are marked with *. Right panel, proliferation of the cells was measured by cell proliferation (MTS) assay after 3 days of treatment of cell variants: MDA-MB-435 shSCR and shB7-H3 cells with 2 μM API-2 (upper panel, *p = 0.0008) and MDA-MB-231 shSCR and shB7-H3 cells with 2 μM API-2 (middle panel, *p = 0.0005) and 200 nM everolimus (bottom panel, *p = 0.0053). ± S.D., statistically significant results are marked with *. All data were normalized, and relative to untreated cells. (**B**) Cell confluence-based growth curves were measured growing the cells in IncuCyte FLR or IncuCyte ZOOM Kinetic Imaging System (Essen BioScience). Cells were scanned every three-hour during the times indicated. The data is presented as percent cell confluence ± S.D. To facilitate comparisons, data from API-2 (middle panel) and everolimus (bottom panel) are shown in two separate plots, which include the same set of data from shSCR and shB7-H3 cells. Cell variants and conditions are as in A, right panels. (**C**) Cell confluence based growth curves was measured of parental MDA-MB-435 cells with 2 μM API-2 and parental MDA-MB-231 cells with 2 μM API-2 and 200 nM everolimus, with or without the presence of 100 ng/ml B7-H3 monoclonal inhibitory antibody (α-B7-H3) (BRCA84D). The data is presented as percent cell confluence ± S.D. (**D**) Immunoblot of B7-H3 and tubulin expression from total cell lysates from MDA-MB-435 shSCR and shB7-H3 cells with and without 2 μM API-2 for 24 h (left panels), and MDA-MB-231 shSCR and shB7-H3 cells with or without 2 μM API-2 and 200 nM everolimus for 24 h (right panels). Plots show quantified immunoblot bands from B7-H3/tubulin, in arbitrary units (AU) ± S.D. In all experiments (A, B, C and D) DMSO was used as a vehicle control.

**Figure 3:**
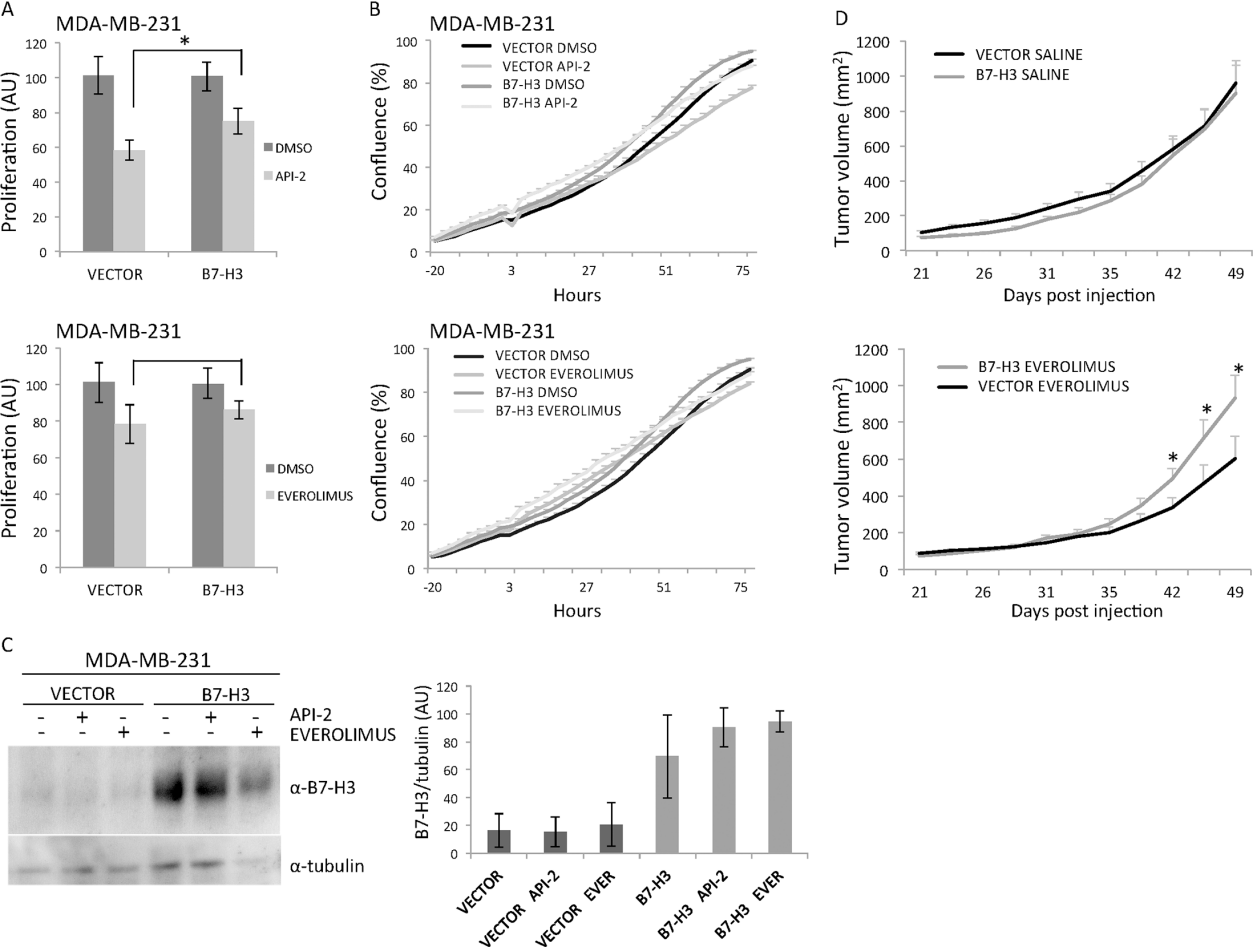
*In vitro* and *in vivo* effects of MDA-MB-231 overexpressing B7-H3 cells treated or not with API-2 and everolimus (**A**) Proliferation of MDA-MB-231 control (VECTOR) and B7-H3 overexpressing (B7-H3) cells was measured by MTS assay after 3 days of treatment with 2 μM API-2 (left panel, *p = 0.0463) and 200 nM everolimus (right panel, *p* = 0.4528) ± S.D., statistically significant results are marked with *. (**B**) Cell confluence-based growth curves were measured growing the cells in IncuCyte ZOOM Kinetic Imaging System (Essen BioScience). Cells were scanned every three-hour during the times indicated. The data is presented as percent cell confluence ± S.D. To facilitate comparisons, data from API-2 (top panel) and everolimus (bottom panel) are shown in two separate plots, which include the same set of data from Vector and B7-H3 cells. Cell variants and conditions are as in A. (**C**) Immunoblot of B7-H3 and tubulin expression from total cell lysates from MDA-MB-231 VECTOR and B7-H3 cells with or without 2 μM API-2 and 200 nM everolimus treatment for 24 h. Plots show quantified immunoblot bands from B7-H3/tubulin, in arbitrary units (AU) ± S.D. (**D**) Effects of B7-H3 overexpression in MDA-MB-231 xenografts. Growth curves of breast cancer xenografts in nude mice established by s.c injection of 2 × 10^6^ MDA-MB-231 VECTOR and B7-H3 cells in both flanks of nude mice treated by gavage with everolimus (5 mg/kg) or solvent (left and right panel, respectively). The drug/solvent was given at day 21 when the mean tumor diameter was between 5–6 mm, and the tumor diameter was measured 2–3 times per week. Each group consisted of 10 animals and the data is presented as average tumor volume ± S.E.M. (all the *p*-values at each time point after day 42 were significant between the two treated groups, and *p-values were between 0.0212 and 0.0363). In all experiments (A, B, C and D) DMSO was used as a vehicle control.

The B7-H3 knockdown and control cell variants were screened for cell viability using a library of 22 compounds. The screening revealed that several drugs showed significantly different efficacy in B7-H3 knockdown compared to control cells in both the MDA-MB-435 and MDA-MB-231 cell lines. All drug concentrations and relative drug responses are listed in Supplementary Table S1. Interestingly, two small molecule inhibitors targeting the PI3K/AKT/mTOR pathway: API-2 (Triciribidine, AKT inhibitor) and everolimus (mTOR inhibitor) showed a weak, though significant, enhanced growth inhibitory effect in B7-H3 knockdown cells (shB7-H3), compared to the control cells (shSCR) (Figure 2A). This was observed using a) cell viability assay in the drug screening (CTG, Figure 2A, left panels); b) cell proliferation assay (MTS, Figure 2A, right panels); and c) cell growth assay (measured as cell confluence, Figure 2B and Supplementary Figure S2A). Furthermore, overexpression of B7-H3 diminished the inhibitory effect on proliferation (Figure 3A) and cell confluence (Figure 3B and Supplementary Figure S2C).

We tested if we could see similar effect on cell growth of parental MDA-MB-435 and MDA-MB-231 cells by targeting B7-H3 using an inhibitory anti-B7-H3 monoclonal antibody (BRCA84D). As shown in Figure 2C, both parental MDA-MB-435 and MDA-MB-231 cell confluence were reduced in the presence of the anti-B7-H3 (α-B7-H3), similar to that of knocking down B7-H3 (Figure 2B). Additionally, α-B7-H3 pre-treated cells showed significant enhanced growth inhibitory effect of API-2 and everolimus, compared to the control treated cells (Figure 2C and Supplementary Figure S2B).

Immunoblot analysis showed that the protein expression levels of B7-H3 did not change after drug treatment of the cell variants (Figures 2D and 3C). Western blot band intensities are shown in panels next to the blots. Neither of the two drugs induced changes in the cellular localization of B7-H3 assessed by immunofluorescence (data not shown).

### Increased expression of B7-H3 confers resistance of breast cancer cells to everolimus *in vivo*

Next, we examined whether the observed *in vitro* effects of the inhibitors could be confirmed *in vivo*. MDA-MB-231 B7-H3 knockdown, overexpression, and control cells were injected subcutaneously (s.c.) in nude mice. The animals were treated with everolimus (5 mg/kg, three times a week for the first three weeks and thereafter twice a week for 4 weeks) administered by gavage when the tumors had reached a mean diameter of 5 to 6 mm. In MDA-MB-231 shB7-H3 cells, the tumor inhibitory effect of knocking down B7-H3 by itself impeded the analysis of drug effects in these animals (data not shown). Overexpression of B7-H3 by itself did not change the *in vivo* growth rate of the cells (Figure 3D, top panel). Everolimus showed a clear antitumor effect in the mice carrying MDA-MB-231 control vector xenografts. In the B7-H3 overexpressiong xenografts, however, no significant effect of everolimus was seen (Figure 3D, bottom panel). These results are in line with the *in vitro* data. We did not see changes in the morphology of the tumors. The difference in average tumor volume (mm^2^) of the treated versus control xenografts assessed at each time point became statistically significant from day 42 until the end of the experiment (*p values at each time point were between 0.0212 and 0.0363). Together the results strengthen the conclusion that B7-H3 plays an important role in the sensitivity of breast cancer cells to everolimus.

We also tested MDA-MB-435 B7-H3 knockdown and control cells *in vivo*, by s.c. injection in nude mice treated intraperitoneally with API-2 (5 mg/kg, twice a week). However, in this experimental setting we did not see any inhibitory effect of API-2 on tumor growth *in vivo* (data not shown).

### Effect of B7-H3 expression on the modulation of AKT/mTOR/p70S6K pathway by API-2 and everolimus

**Figure 4:**
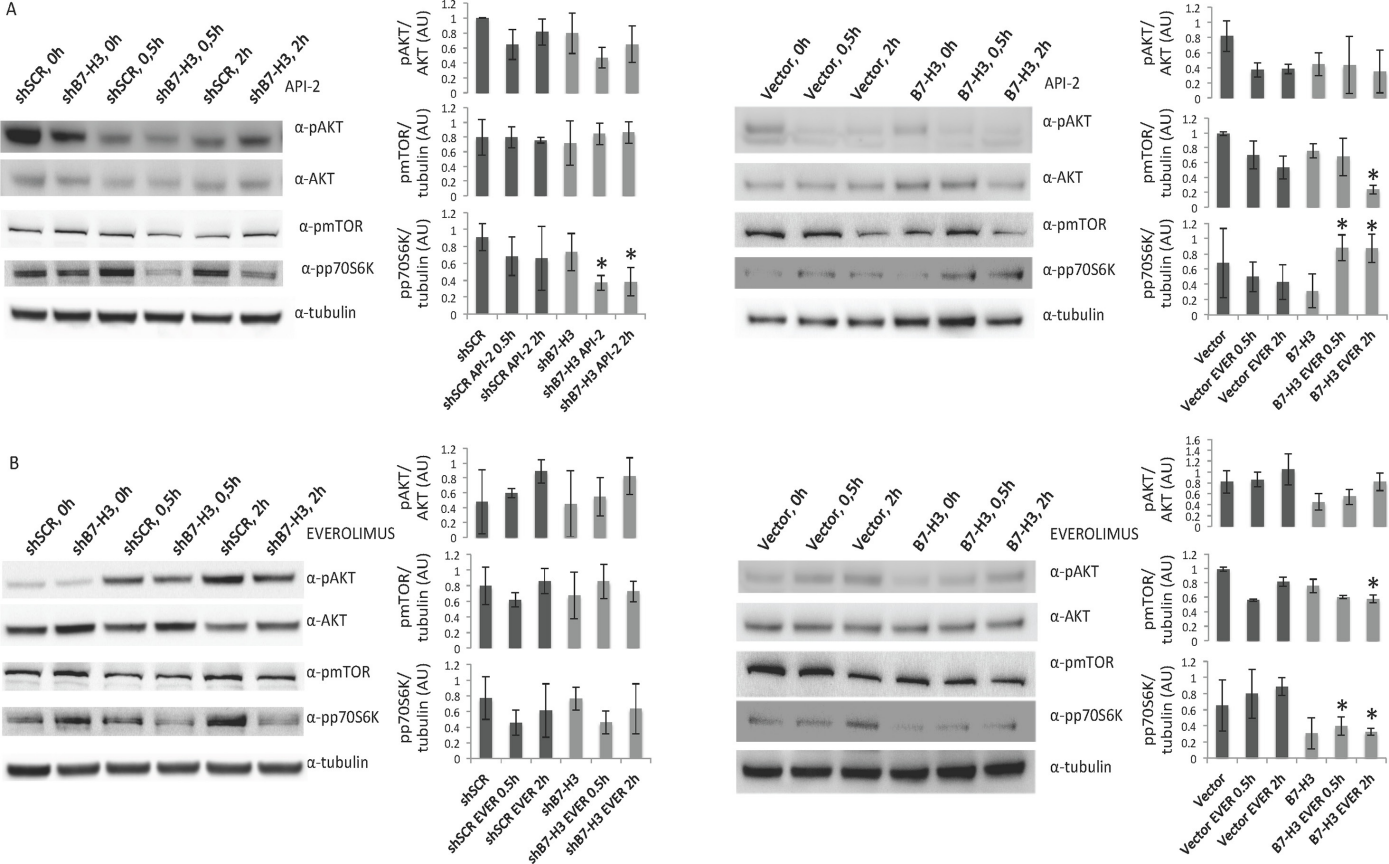
Immunoblot analysis of AKT, mTOR and p70S6K activation in MDA-MB-231 cell variants treated or not with API-2 and everolimus Immunoblot of phospho AKT (pAKT), AKT, phospho-mTOR (pmTOR), phospho-p70S6K (pp70S6K) and tubulin expression from total lysates from (**A**) MDA-MB-231 control (shSCR) and knockdown B7-H3 (shB7-H3) cells (left panels), Vector control and B7-H3 overexpressing cells (right panels) treated or not with 2 μM API-2 for 0,5 h and 2 h. (**B**) MDA-MB-231 shSCR and shB7-H3 cells (left panels) and Vector and B7-H3 cells (right panels) treated or not with 200 nM everolimus for 0, 5 h and 2 h. Plots show quantified immunoblot bands from pAKT/AKT, pmTOR/tubulin and pp70S6K/tubulin, in arbitrary units (AU) ± S.D. Significant changes are shown with *p < 0.01. In all experiments (A and B) DMSO was used as a vehicle control.

We looked further into the mechanism of the observed effects and analyzed the phosphorylation status of components of the AKT/mTOR/p70S6K pathway by immunoblot analysis. We did not detect significant changes in the phosphorylation levels of the AKT/mTOR components upon knocking down B7-H3. However, we observed slightly decreased phosphorylation levels of AKT in B7-H3 overexpressing cells (Figure 4A and 4B) left panels.

In API-2 and everolimus treated B7-H3 overexpressing cells phospho-mTOR levels were decreased (Figure 4A and 4B, right panels). In addition, MDA-MB-231 shB7-H3 cells treated with API-2 showed a significant reduction in phospho-protein 70 S6 kinase (p70S6K), a downstream target of mTOR (Figure 4A, left panel), whereas API-2-treated MDA-MB-231 B7-H3 overexpressing cells showed a significantly increased phospho-p70S6K level (Figure 4A, right panel). However, in everolimus-treated MDA-MB-231 cells we observed decreased phospho-p70S6K levels in B7-H3 overexpressing cells compared to the B7-H3 knockdown and vector cells (Figure 4B, right panel). Western blot band intensities are shown in panels next to the blots, and significant changes are shown with an *.

Interestingly, treatment with everolimus increased the levels of phospho-AKT in both MDA-MB-231 cell variants (Figure 4B, left and right panels). These results are in consistence with previous findings suggesting a positive feedback activation of AKT by everolimus [26]. The phosphorylation levels of several other central proteins in this pathway, including elF4E, GSK-3β and BAD, did not show any differences between the cell variants (data not shown).

### API-2 and everolimus reduce the glycolysis of breast cancer cells

**Figure 5:**
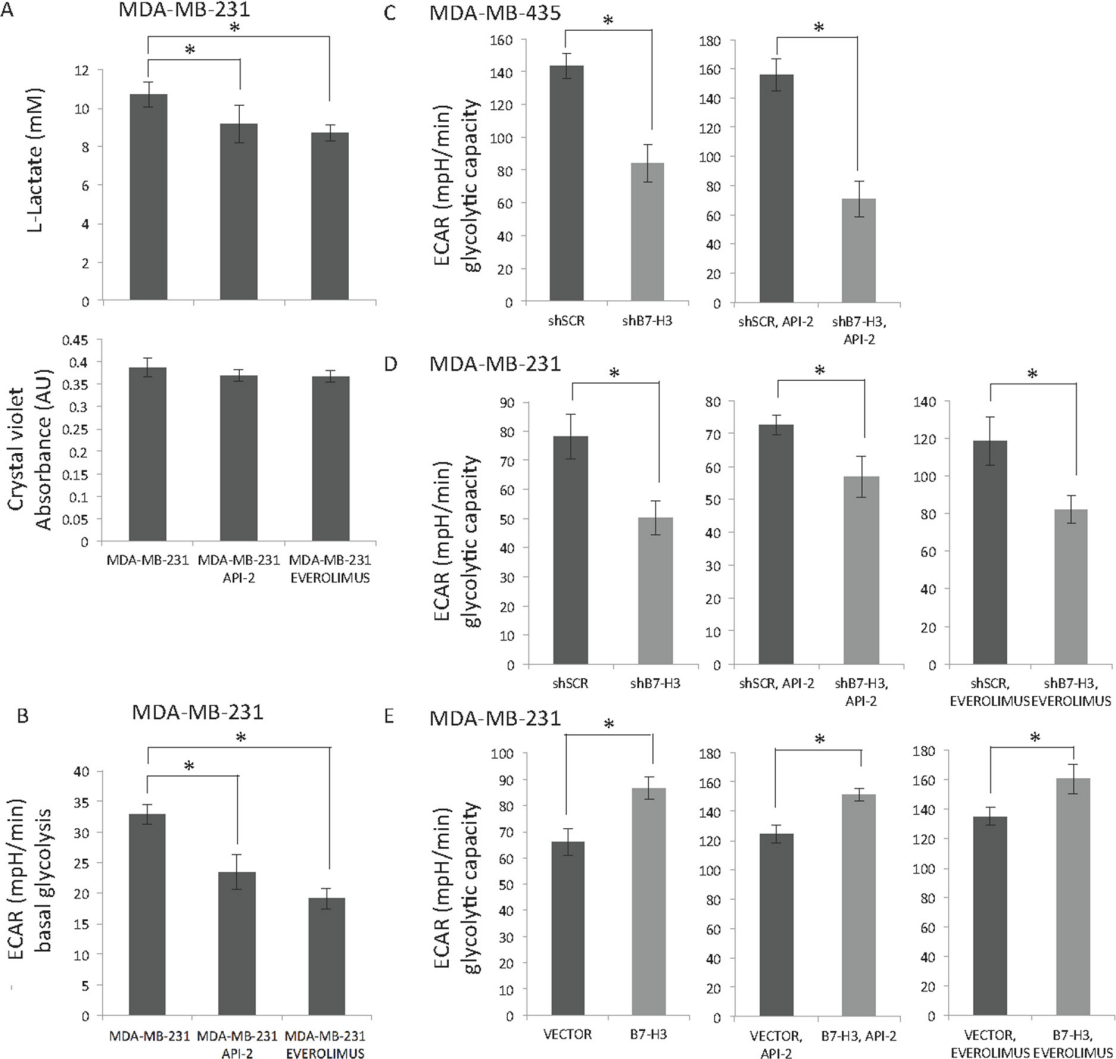
Effect of API-2, everolimus and B7-H3 on glycolysis in breast cancer cells (**A**) Lactic acid levels were measured in MDA-MB-231 cells (upper panel) treated or not with 2 μM API-2 and 200 nM everolimus for 24 h ± S.D., statistically significant results are marked with *: API-2: *p = 0.0469, everolimus: *p = 0.0051). Bottom panel show cell number measured by crystal violet staining. (**B**) Seahorse Extracellular Flux Analyzer XF96e was used to measure the Extracellular Acidification Rate (ECAR) in MDA-MB-231 cells (in B-E ± S.D.). Basal glycolysis in MDA-MB-231 cells with API-2 inhibitor (*p < 0.0001), and everolimus (*p = 0,0008). (**C**) Glycolytic capacity of shSCR and shB7-H3 MDA-MB-435 cells was measured (left panel, *p = 0.000313), with API-2 treatment (2 μM, right panel, *p = 1.87E-07). (**D**) Glycolytic capacity of shSCR and shB7-H3 MDA-MB-231 cells was measured (left panel, *p = 0.003365), with API-2 treatment (2 μM, middle panel, *p = 0.00652), and with everolimus (200 nM, right panel, *p = 0.002546). (**E**) Glycolytic capacity of Vector and B7-H3 MDA-MB-231 cells was measured (left panel, *p = 0.000833), with API-2 treatment (2 μM, middle panel, *p = 0.000395), and with everolimus (200 nM, right panel, *p = 0.004456). In all experiments (A, B, C, D and E) DMSO was used as a vehicle control.

We did not observe differences in cell cycle distribution or cell death by apoptosis nor necrosis in the B7-H3 cell variants upon treatment with the API-2 and everolimus inhibitors (data not shown), and looked therefore into other mechanistic explanation of the observed antiproliferative effect. Since members of AKT/mTOR/p70S6K pathway is known to be master regulators of aerobic glycolysis in normal tissue as well as in cancer cells [27, 28], we wanted to test whether the two inhibitors had any effect on metabolism. Hence, we measured the L-lactate levels in cell media as a readout of the intracellular glycolytic activity. We observed significantly reduced L-lactate levels in MDA-MB-231 cells upon treatment with both API-2 and everolimus (Figure 5A, upper panel). The same wells were stained with crystal violet, and show equal amount of cells in all conditions (Figure 5A, lower panel).

Further ExtraCellular Acidification Rate (ECAR) was measured as a readout of the basal glycolysis using the Seahorse technology, and confirmed that both API-2 and everolimus decreased basal glycolysis in MDA-MB-231 cells (Figure 5B). Together, these results show that API-2 and everolimus decrease the intracellular basal glycolysis in MDA-MB-231 cells, as has been previously found in other cancer cells [29, 30]. In MDA-MB-435 cells treated with API-2 we observed slightly lower L-lactate and ECAR levels, yet not significant (data not shown).

### Decreasing expression of B7-H3 reduces glycolytic capacity of breast cancer cells treated with API-2 and everolimus

Next, we analyzed the effect of B7-H3 expression on glycolysis, and in MDA-MB-435 and MDA-MB-231 B7-H3 knockdown cells the glycolytic capacity was decreased (Figure 5C and 5D, left panels), while cells with overexpressed B7-H3 had higher glycolytic capacity (Figure 5E, left panel). Importantly, as shown in Figure 1B and 1C, overexpressing B7-H3 in MDA-MB-231 cells had weak impact on cell proliferation, suggesting that the effect of B7-H3 on glycolytic capacity is independent of the proliferative rate of the cells.

Since both the inhibitors and decreased B7-H3 expression reduced the basal glycolysis and glycolytic capacity, respectively, we tested if we could observe an additive effect on the glycolytic capacity. Upon treatment with API-2 the glycolytic capacity in shSCR MDA-MB-435 cells was higher than in the shB7-H3 cells (Figure 5C, right panel). Also, the glycolytic capacity in API-2- or everolimus-treated MDA-MB-231 shSCR cells was higher than in treated shB7-H3 cells (Figure 5D, middle and right panels). The opposite effect was observed in the B7-H3 overexpressing cells where glycolytic capacity was increased compared to the control vector cells (Figure 5E, middle and right panels). Together, these results demonstrate that B7-H3 knockdown decreases glycolytic capacity in breast cancer cells, in addition to the decrease in glycolysis induced by API-2 and everolimus. These results suggest independent mechanisms of inhibition of glycolysis, supporting the notion that a combinatorial approach of inhibiting both B7-H3 and glycolysis (e.g. mTOR/AKT/p70S6K) pathway would be beneficial.

## DISCUSSION

B7-H3 is highly expressed in various breast cancer cell lines and tumors, including in TNBC [18–22], correlating with cancer progression and poor outcome. In addition, B7-H3 expression has also been linked to resistance to chemotherapeutic agents [17, 31]. Here, we performed a compound screen using human TNBC cell lines and found that cell responsiveness to API-2 (triciribidine) and everolimus (RAD-001), two inhibitors targeting proteins in the AKT/mTOR pathway, was increased under conditions with low B7-H3 expression. We observed a weak, but significant increase in the *in vitro* antiproliferative effect of API-2 and everolimus in B7-H3 knockdown cells, as well as in anti-B7-H3 antibody treated cells. The effect of B7-H3 expression on drug response was further strengthened by *in vivo* results showing significantly reduced effect of everolimus on B7-H3 overexpressing MDA-MB-231 xenografs.

To our knowledge, this is the first time that B7-H3 is linked to decreased sensitivity to targeted therapy with small molecule inhibitors. Inhibitors of proteins in the AKT/mTOR pathway, including everolimus, are currently being tested in the clinic as new therapeutic agents in breast cancer patients [32–34]. Our results suggest a link between B7-H3 expression and sensitivity to API-2 and everolimus inhibitors. In this regard, a recent report on non-small cell lung cancer relates poor prognosis to the up-regulation of B7-H3 by ILT-4 through PI3K/AKT/mTOR pathway [35]. Our analysis on the phosphorylation status on proteins from the PI3K/AKT/mTOR pathway indicates a differential effect on mTOR and the mTOR-downstream target p70S6K. This suggests a different B7-H3-mediated mechanism of API-2 and everolimus on breast cancer cell sensitivity downstream of mTOR.

B7-H3 expression did not alter activation of the AKT/mTOR pathway, but the high B7-H3 expression levels in the tumor cells increased cell survival and glycolysis (Supplementary Figure S3, left panel). B7-H3 expression was not altered by the presence of API-2 or everolimus inhibitors, but their inhibitory effect on both cell growth and glycolysis was dependent on the endogenous expression levels of B7-H3. Inhibition was enhanced by B7-H3 knockdown or antibody targeting, while increased levels of B7-H3 counteracted this inhibition (Supplementary Figure S3, right panel). Together, these results suggest that API-2 and everolimus inhibition together with B7-H3 knockdown would act in concert by putative different mechanisms.

Several B7 family members have emerged as feasible therapeutic targets in cancer immunotherapy [36–38], including B7-H3 in breast cancer [39, 40]. Regardless of its regulatory immunological functions, non-immunological roles for B7-H3 in tumor biology have also been identified. We have shown that B7-H3 expression correlated with increased migration and invasion *in vitro* [25], which was confirmed by *in vivo* studies showing a decreased metastatic potential in nude mice injected with B7-H3 knockdown cancer cells [12]. In addition, we reported a connection between the expression of B7-H3 and the resistance to the chemotherapeutic drug Paclitaxel in breast cancer cells [17]. These results, together with our current findings on the B7-H3-mediated resistance to small molecule inhibitors, may indicate a more general association between B7-H3 expression and drug resistance in cancer. Hence, we speculate that B7-H3 protein could be both a prognostic marker and a potential therapeutic target.

The B7-H3 dependent effect on API-2- and everolimus-sensitivity seems to be linked to the glycolytic capacity of the cells. Thus, we observed decreased glycolysis in the tumor cells treated with API-2 or everolimus, in line with recent reports in other human cancer cells, as well as with the connection between AKT/mTOR/p70S6K signaling in cancer and glycolysis [29, 30, 41]. Moreover, we found that B7-H3 increased the glycolytic capacity of TNBC cells. These changes linked to B7-H3 expression are not exclusive to breast cancer cell lines as similar results were obtained with melanoma cell lines (data not shown). The molecular basis of the link between cell metabolism and drug resistance mediated by B7-H3 needs to be explored. Interestingly, we have previously found a correlation between B7-H3 expression and STAT3 signaling, a protein that has emerged as a master regulator also of glycolysis [42].

In summary, our results show that B7-H3 expression confers resistance of breast cancer cells to anticancer therapies. In addition, we have unveiled a role for B7-H3 in cell metabolism. Further identification of signaling molecules/pathways and metabolic regulators influenced by B7-H3 will be important in defining the role of B7-H3 in glycolysis, drug resistance and cancer metastasis.

## MATERIALS AND METHODS

### Cell culture, plasmids, immunoblot, antibodies and reagents

MDA-MB-435 and MDA-MB-231 cell variants were grown in Dulbecco's Modified Eagle Medium (DMEM; Invitrogen) supplemented with 10% FBS, and 2 mM L-glutamine. The mammalian expression plasmid to generate stable cell lines pCDNA6-B7-H3, was obtained by subcloning from pIRES-B7-H3 cut with NheI/SacII (NEB), and stable cell lines were selected with 10 μg/ml Blasticidin (Gibco) for three weeks. Whole cell protein extracts were prepared by total cell lysis and Western blots were preformed as described previously in [19, 43]. Antibodies used were: B7-H3 (AF1027, R&D), tubulin (CP06, Millipore), and pAKT (#4060), AKT (#9272), pmTOR (#2971) and pp70S6K (#9206) (all from Cell Signaling). B7-H3 monoclonal antibody (BRCA84D) was kindly provided by MacroGenics. Protein concentrations from total cell lysates was measured using The Pierce^®^ BCA Protein Assay Kit (Thermo Scientific, USA). API-2 (Triciribidine, Sigma-Aldrich), and everolimus (RAD-001, Sigma-Aldrich and Invivogen) were used at different concentration, during the indicated times. DMSO was always used as a control, at the same final concentration as in resuspended and diluted drugs. Western blot band intensities were quantified using ImageJ software from two-three independent biological experiments ± S.D.

### Drug screening

The cell lines were screened for a library of 22 compounds (Supplementary Table S1). The compounds were printed in seven dilutions (0.34 pM – 20 μM) with two technical replicates using a Hamilton robot (Hamilton Robotics Inc., Reno, NV, USA) in 384-well plates (Corning Inc., NY, USA). The cell lines MDA-MB-435 (shSCR and shB7-H3) and MDA-MB-231 (shSCR and shB7-H3) were seeded in 384-well plates using 500–900 cells per well. Cells in suspension were dispensed to each well by a Multidrop Reagent dispenser Combi (Thermo Fischer Scientific, USA). The screening was conducted for five days, and cell viability was measured using CellTiter-Glo^®^ assay (CTG, Promega Corp, Madison, WI, USA) where luminescence signal was read by a MicroBeta TriLux (PerkinElmer, Waltham, MA, USA). The drug screening was performed twice for each cell line variant, and the reproducibility of the duplicate screens was evaluated with respect to their correlation in a scatter plot (Supplementary Figure S4). A high correlation, ranging from 0.87–0.91, showed that the replicate screens were similar and thus biologically representative. Cell viability was converted into relative response using the average of the lowest concentrations of all compounds as control. The relative response was further corrected through compound-wise normalization to the average response of the three lowest concentrations. Drug responses were plotted in GraphPad Prism version 5.0 (GraphPad Software, San Diego, CA, USA) and IC_50_ (half maximal inhibitory concentration) values were obtained for compounds that showed effect. The EC_50_ values of API-2 and everolimus in the cell variants are presented in Supplementary Table S2.

### *In vitro* proliferation and cell confluence assay

Cells (1 × 10^4^ cells) were plated in 96-well culture plates in media with or without API-2 or everolimus and incubated for indicated time points. Cell proliferation was determined by CellTiter 96 Aqueous One Solution Cell Proliferation Assay Kit (MTS, Promega Corp, Madison, WI, USA). Cells were processed 72 h post-treatment. Absorbance was measured at 490 nm using Wallac Victor2 1420 Multilabel Counter (PerkinElmer, USA). The data is presented as the average absorbance ± S.D. corrected for background from one representative experiment. Cell proliferation assays were performed in at least triplicate wells three times for each cell line at separate days. Cell confluence was measured growing the cells in IncuCyte FLR or IncuCyte ZOOM Kinetic Imaging System (Essen BioScience) that estimate cell growth and number. Cells were scanned every three-hour during the times indicated. The data is presented as cell confluence ± S.D.

### *In vivo* studies

Four groups of 10 female Balb/c nude mice, bred and used at the nude rodent facility at the Oslo University Hospital, The Norwegian Radium Hospital. The animals were maintained under specific pathogen-free conditions, and food and water were supplied ad libitum. Animal experiments were done according to protocols approved by the animal care and use committee and were in compliance with the guidelines on animal welfare of the Norwegian National Committee for Animal Experiments. When the animals were 6 to 8 weeks of age, 2 × 10^6^ cells in 0.2 mL PBS were injected s.c. in both flanks of nude mice. For therapy experiments, a stock solution of everolimus in DMSO (28 mM) was dissolved in 0.5% methylcellulose and when the mean tumor diameter was between 5 to 6 mm 5 mg/kg of the drug was administered by gavage three times a week for the first three weeks and thereafter twice weekly. The control mice received only the DMSO in 0.5% methylcellulose (Sigma-Aldrich). Tumor diameters were measured 2–3 times per week. Tumor volume was calculated by the formula 0.5 × length × width^2^ and growth curves constructed, and the data is presented as average tumor volume ± S.E.M.

### Extracellular lactic acid levels and acidification rate

Glycolysis Cell-Based Assay Kit (Cayman chemicals) was used to measure extracellular L-lactic acid levels according to manufacturer's instructions. Cell number was measured by crystal violet (0.05%) staining, resuspended in 1% SDS. Luminescence was measured at 490 nm using Wallac 1420 (PerkinElmer, USA) with the MicroBeta Windows Workstation (PerkinElmer, USA) ± S.D. XF96 Glycolysis stress test was performed using Seahorse Extracellular Flux Analyzer XF96e to measure the ExtraCellular Acidification Rate (ECAR) according to manufacturer's instructions. Cells were seeded in Seahorse plate 48 hours after splitting, and cultured overnight to 80% confluence. One hour before measurement, the culture medium was replaced with cellular assay medium (Seahorse Bioscience) supplemented with 2 mM glutamine, and incubated for 1 hour in a CO_2_-free incubator. And assay was performed according to Seahorse protocols with the final concentrations of 10 mM glucose, 1 μM of oligomycin and 100 mM of 2-Deoxy-D-glucose (2-DG). Seahorse assays were performed in at least triplicate wells in three independent experiments for each cell line and condition at separate days ± S.D.

## SUPPLEMENTARY MATERIALS FIGURES AND TABLES




