# Supplementary material for: Decreased expression of B7-H3 reduces the glycolytic capacity and sensitizes breast cancer cells to AKT/mTOR inhibitors
Source: Oncotarget. 2016 Jan 12;7(6):6891–901. doi: 10.18632/oncotarget.6902 (PMC4872756; doi:10.18632/oncotarget.6902)
Supplement: Supplementary file 1 [file oncotarget-07-6891-s001.pdf]

Decreased expression of B7-H3 reduces the glycolytic capacity and sensitizes breast cancer cells to AKT/mTOR inhibitors

Supplementary Materials

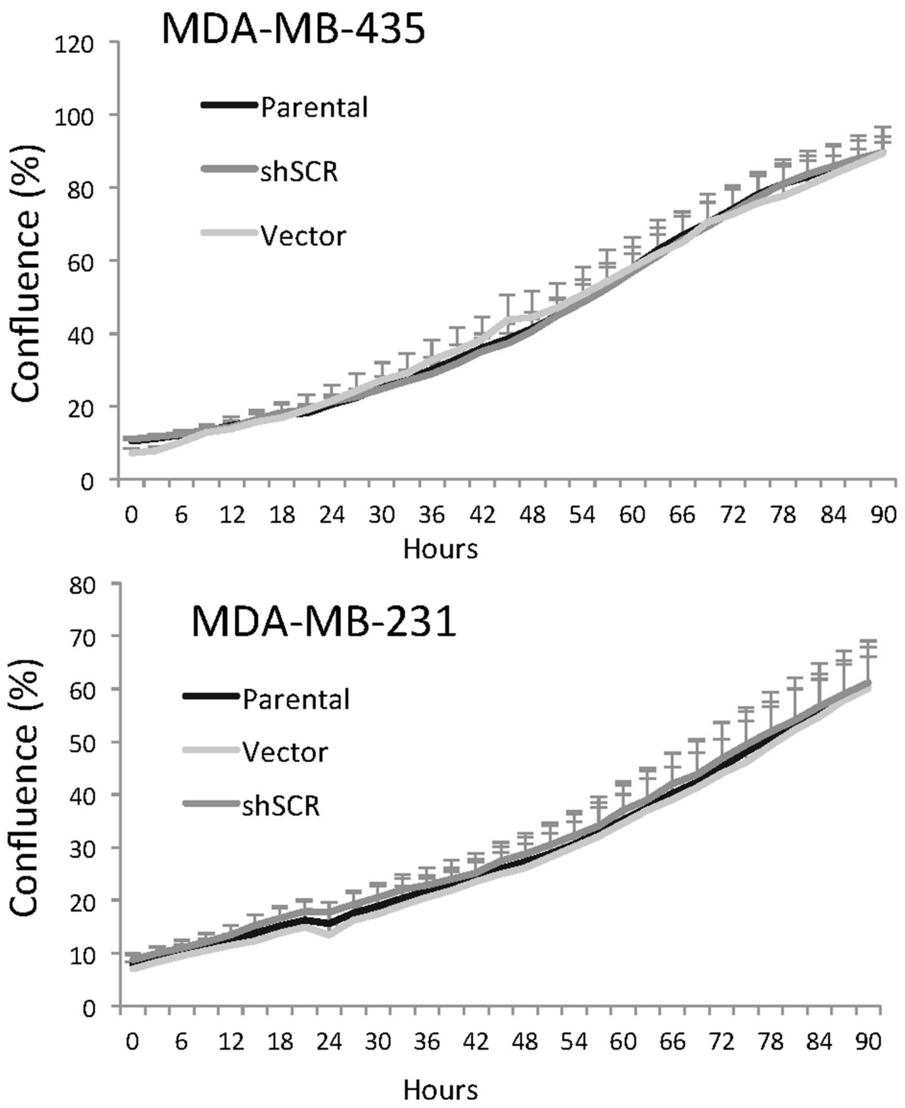

Supplementary Figure S1: Cell confluence based growth curves of the MDA-MB-435 and MDA-MB-231 parental, vector and shSCR cell variants were measured growing the cells in IncuCyte FLR or IncuCyte ZOOM Kinetic Imaging System (Essen BioScience). Cells were scanned every three-hour during the times indicated. The data presented as percent confluence ± S.D.

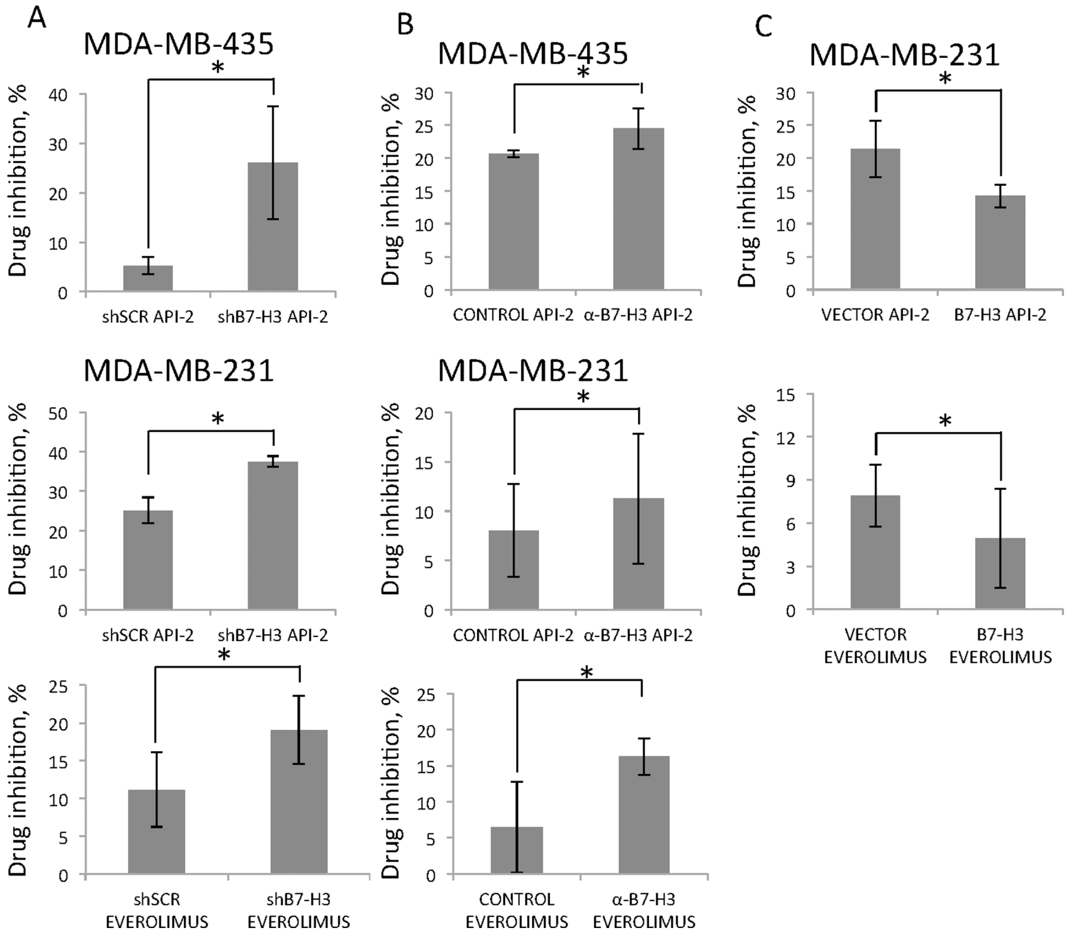

Supplementary Figure S2: Relative confluence shown as % drug inhibition from two-three independent experiments is shown. (**A**) MDA-MB-435 B7-H3 knockdown (shB7-H3) and control (shSCR) cells treated with API-2 for 99 h (*p = 0.000376). Middle and bottom panel) MDA-MB-231 shB7-H3 and shSCR cells treated with API-2 for 99 h (*p = 1.71109E–05) or everolimus for 75 h (*p = 9.65929E–05). (**B**) MDA-MB-435 parental pre-treated cells with or without 100 ng/ml B7-H3 monoclonal inhibitory antibody (α-B7-H3) (BRCA84D), and treated with API-2 for 99 h (*p = 0.005385). Middle and bottom panel) MDA-MB-231 parental control and α-B7-H3 cells treated with API-2 for 99 h (*p = 0.020465) or with everolimus for 99 h (*p = 0.001299). (**C**) Top and bottom panel) MDA-MB-231 control vector and B7-H3 cells treated with API-2 for 99 h (*p = 0.0404643) or everolimus for 99 h (*p = 0.0270342).

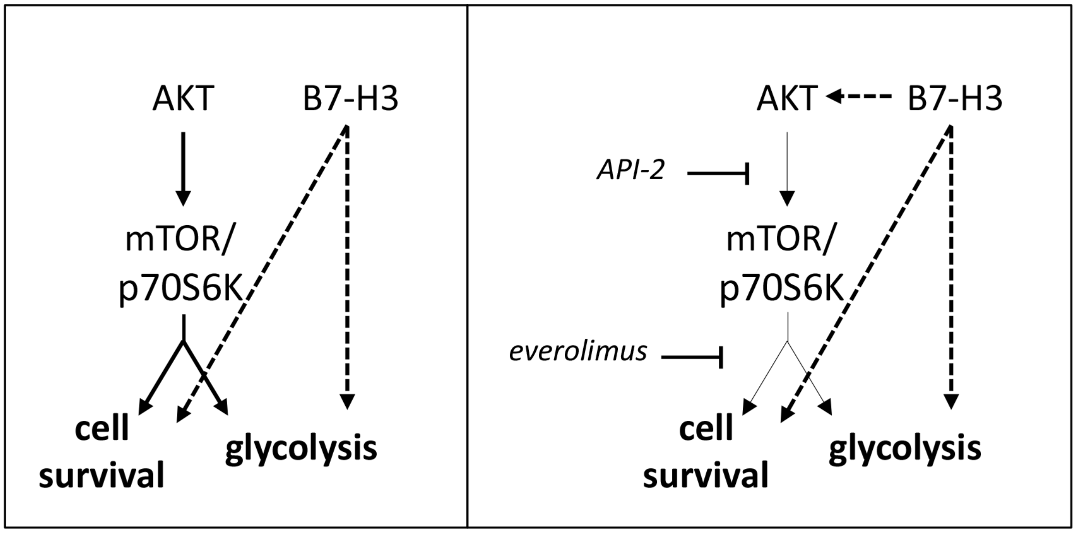

Supplementary Figure S3: Schematic of B7-H3 role in the control of cell survival and glycolysis in breast cancer cells. In the absence of API-2 or everolimus inhibitors (left panel), B7-H3 expression did not affect AKT/mTOR/p70S6K pathway activation, but enhanced cell survival and glycolysis. In the presence of API-2 or everolimus (right panel), low expression levels of B7-H3 increased the inhibitory effect of API-2 and everolimus on cell survival and glycolysis, whereas high levels of B7-H3 counteracted the effect of the inhibitors. This suggests that, in the presence of API-2 or everolimus inhibitors, B7-H3 facilitates AKT/mTOR/p70S6K activation, producing an additive positive effect on cell survival and glycolysis.

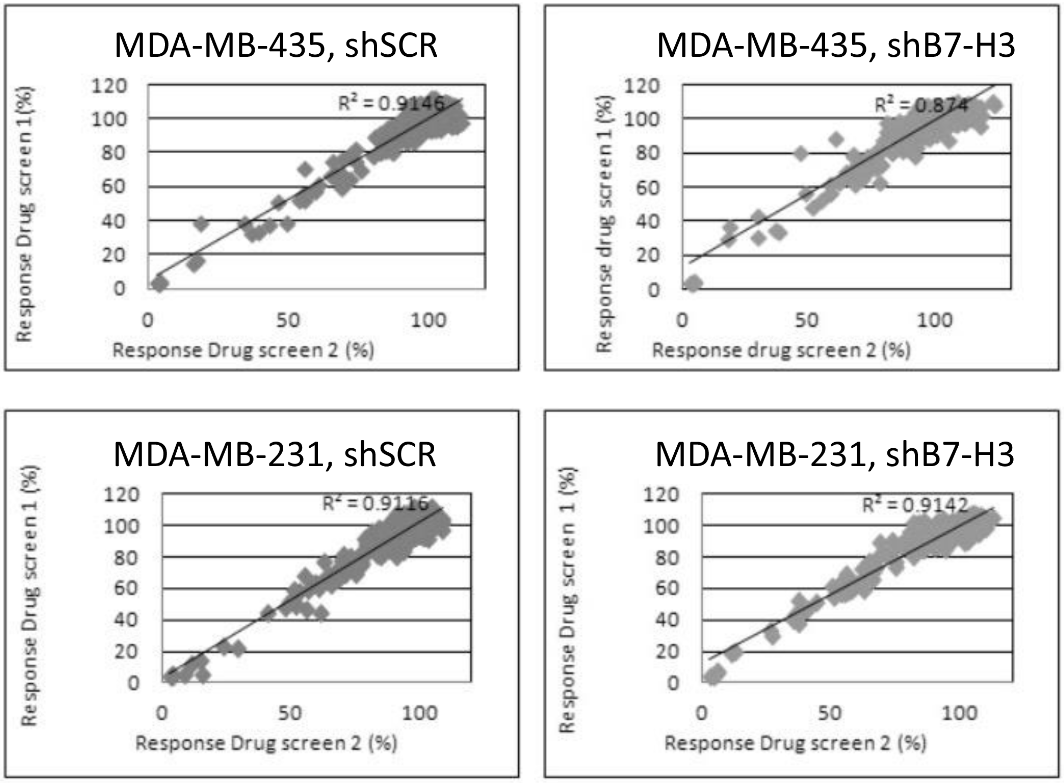

Supplementary Figure S4: Scatter plots of two independent drug screening experiments for each cell line; MDA- MB-435 B7-H3 knockdown (shB7-H3) and control (shSCR) cells and MDA-MB-231 shB7-H3 and shSCR cells. Satisfactory correlation between duplicate drug screens was observed. All data were normalized before the correlation was examined.

Supplementary Table S1: Relative growth response after 5 days of treatment with the 22 different anticancer drugs. All drugs and concentrations are indicated. Relative values are shown for all concentrations of all drugs in MDA-MB-435 and MDA-MB-231 control cells (shSCR) and B7-H3 knockdown (shB7-H3). All data were normalized.

Supplementary Table S2: The EC_{50} Values of cell variants; MDA-MB-435 B7-H3 knockdown (shB7-H3) and control (shSCR) cells and MDA-MB-231 shB7-H3 and shSCR cells

	EC50 Values (μM)			
	MDA-MB-435		MDA-MB-231	
	shSCR	shB7-H3	shSCR	shB7-H3
API-2	2,61	1,51	3,03	1,06
Everolimus	–	–	0,02	0,01

A square with a dash (–) indicate that there was no significantly different efficacy between the cell line variants, and EC_{50} values was thus not calculated.
